# Case Report: Severe QL1706-associated immune-related inflammatory myopathy presenting with respiratory dysfunction, bulbar weakness, ocular motility impairment, and suspected cardiac involvement

**DOI:** 10.3389/fonc.2026.1851665

**Published:** 2026-08-21

**Authors:** Jie Yu, Danye Qian, Cong Wang, Fengming Zheng

**Affiliations:** Department of Geriatrics, Third People’s Hospital of Hangzhou, Hangzhou, China

**Keywords:** immune-related adverse events, immune-related inflammatory myopathy, ocular motility impairment, QL1706, suspected cardiac involvement

## Abstract

QL1706 is a novel bifunctional antibody targeting PD-1 and CTLA-4 that has received conditional approval in China for cervical cancer. Its full immune-related adverse event profile remains incompletely defined. Here, we report a 61-year-old woman with endometrial clear cell carcinoma who developed severe immune-related inflammatory myopathy 4 weeks after the first cycle of QL1706 combined with paclitaxel and carboplatin. She presented with dyspnoea, generalised weakness, dysphagia and ocular motility impairment. Laboratory tests showed marked elevations in creatine kinase, CK-MB, lactate dehydrogenase and cardiac troponin T, and electrophysiological findings supported a predominant myopathic process. The final diagnosis was QL1706-associated severe immune-related inflammatory myopathy with respiratory and ocular involvement and clinically suspected cardiac involvement. Myocarditis, ocular myositis and myositis–myocarditis–myasthenia gravis overlap syndrome could not be definitively confirmed. The patient improved after high-dose methylprednisolone, intravenous immunoglobulin and tacrolimus. This case highlights the need for early recognition, multidisciplinary evaluation and prompt immunosuppressive treatment for severe neuromuscular immune-related adverse events during QL1706-based therapy.

## Introduction

QL1706 is a bifunctional PD-1/CTLA-4 dual-blockade antibody product composed of iparomlimab, an IgG4 anti-PD-1 antibody, and tuvonralimab, an IgG1 anti-CTLA-4 antibody, at a fixed 2:1 ratio (1). The CTLA-4-targeting component, tuvonralimab, was engineered to have a shorter elimination half-life, thereby limiting systemic CTLA-4 exposure and potentially reducing toxicity associated with CTLA-4 blockade. QL1706 has shown promising antitumor activity in cervical cancer, ovarian cancer and other solid tumours (1, 2), and was approved in China for the treatment of cervical cancer (3). However, dual checkpoint blockade is generally associated with a higher risk of immune-related adverse events (irAEs) than ICI monotherapy. In a systematic review, the mean irAE event rate was 45.7% for ICI combination therapy compared with 30.5% for ICI monotherapy. In the first-in-human phase I/Ib study of QL1706, any-grade irAEs and grade ≥3 irAEs occurred in 46.1% and 8.1% of patients, respectively (4, 5).

According to data presented at the European Society for Medical Oncology (ESMO) Congress 2025, the three-year update of the phase II DUBHE-C-204 study reported the efficacy and safety of QL1706 combined with chemotherapy, with or without bevacizumab, in patients with untreated recurrent or metastatic cervical cancer. In the safety analysis, grade ≥ 3 treatment-related adverse events occurred in 75% of patients, whereas grade ≥ 3 irAEs were observed in 15% of the cohort. These data indicate that QL1706-based therapy can be associated with clinically significant immune-mediated toxicity. However, the full spectrum and severity of neuromuscular irAEs may not be fully captured in pooled clinical trial data.

Recent case reports have suggested that QL1706 may induce severe immune-related cardiac and muscular toxicities, including myocarditis, myositis and multi-organ irAEs (6, 7). Here, we report a case of QL1706-associated severe immune-related inflammatory myopathy with respiratory and ocular involvement and clinically suspected cardiac involvement. This case further highlights the need for early recognition and multidisciplinary management of severe neuromuscular and cardiac immune-related toxicities during QL1706-based treatment.

## Case presentation

### Clinical course and diagnostic assessment

A 61-year-old woman was diagnosed with endometrial clear cell carcinoma and underwent total hysterectomy with bilateral salpingo-oophorectomy. Postoperative histopathological examination confirmed endometrial clear cell carcinoma. Immunohistochemistry showed positivity for HNF-1β, Napsin A, AMACR/P504S and CK7, supporting the diagnosis. The tumour measured 1.0 × 0.7 × 0.5 cm and showed myometrial invasion of 1.5 mm, corresponding to 15% of the myometrial thickness. Lymphovascular space invasion and perineural invasion were absent, and all six regional lymph nodes were negative for metastasis (0/6). According to the 2023 International Federation of Gynecology and Obstetrics (FIGO) staging system, the tumour was classified as stage IIC.

After surgery, the patient received adjuvant chemoradiotherapy, including pelvic external beam radiotherapy with concurrent cisplatin-based chemotherapy, followed by systemic chemotherapy with paclitaxel and carboplatin. During the second cycle of systemic chemotherapy, QL1706, a bifunctional PD-1/CTLA-4 dual-blockade antibody product, was administered. Approximately 4 weeks after the first QL1706 infusion, the patient was admitted for further evaluation because of poor appetite, overall weakness and diffuse myalgia. After admission, she developed exertional dyspnoea, which progressed rapidly within 72 hours to severe orthopnoea, respiratory distress, dysphagia, dysarthria, and ocular motility impairment.

Initial laboratory tests showed elevated liver enzymes, including aspartate aminotransferase (AST) of 266 U/L and alanine aminotransferase (ALT) of 308 U/L, as well as an increased C-reactive protein level of 24.4 mg/L. Muscle enzymes and cardiac-associated biomarkers subsequently increased markedly: creatine kinase (CK) rose to 3579 U/L, CK-MB to 97 U/L, and cardiac troponin T (cTnT) to 0.588 ng/mL (normal value, < 0.014 ng/mL). Lactate dehydrogenase (LDH) increased to 985 U/L during the early phase of symptom onset. The earliest available pre-onset LDH value was 220 U/L, which was within the institutional reference range of 120–250 U/L.

After admission, the patient developed nocturnal chest tightness. Electrocardiography showed sinus rhythm with nonspecific ST-T abnormalities and QT prolongation. On day 4 after admission, dyspnoea worsened substantially, and the patient became unable to lie flat and developed orthopnoea. Pulmonary function testing showed reductions in vital capacity and forced vital capacity, with a severe reduction in forced expiratory volume in 1 s, while the FEV1/FVC ratio remained within the normal range. The report interpreted these findings as severe restrictive ventilatory dysfunction. Because of poor patient cooperation, diffusing capacity for carbon monoxide could not be measured. Twenty minutes after inhalation of 400 μg salbutamol, forced vital capacity did not improve from baseline, and forced expiratory volume in 1 s increased by only 6.4% (50 mL), indicating a negative bronchodilator test. Taken together with progressive muscle weakness and dyspnoea, these findings suggested possible respiratory muscle involvement. However, because the test was affected by the patient’s cooperation and respiratory muscle strength or diaphragmatic function was not assessed, respiratory muscle involvement could not be confirmed on the basis of pulmonary function testing alone.

Emergency computed tomography angiography showed no evidence of pulmonary embolism or acute coronary artery disease. Given the patient’s critical condition and the absence of coronary CTA findings suggestive of acute coronary syndrome, invasive coronary angiography was not pursued. Transthoracic echocardiography showed left atrial enlargement, thickening of the mid-to-upper interventricular septum and dilatation of the ascending aorta. However, left ventricular systolic function was preserved, with a left ventricular ejection fraction of 64.2%, and no definite regional wall-motion abnormality or pericardial effusion was observed. Bedside echocardiography also showed no obvious structural or functional cardiac abnormality. Although chest tightness, dyspnoea, ECG abnormalities and elevated CK-MB and cTnT suggested possible cardiac involvement, cTnT elevation may have been partly influenced by skeletal muscle injury in the setting of severe skeletal muscle inflammation. Therefore, the available evidence was insufficient to confirm immune-related myocarditis.

The patient had bilateral lower-limb weakness and pain. Muscle strength was normal in both upper limbs, whereas lower-limb strength was Medical Research Council (MRC) grade 4/5 bilaterally. Electrophysiological assessment included nerve conduction studies, repetitive nerve stimulation and needle electromyography. Repetitive nerve stimulation showed no obvious abnormality. Nerve conduction studies showed reduced motor evoked potential amplitudes in the right median and right common peroneal nerves, whereas sensory nerve conduction studies showed no obvious abnormality. Needle electromyography showed myogenic changes in the right iliopsoas muscle and partial myogenic changes in the right vastus medialis, deltoid and biceps brachii muscles, accompanied by partial neurogenic changes. Overall, the electrophysiological impression was predominantly progressive myopathy. No dermatomyositis-like rash was observed during the clinical course.

On day 13 after admission, the patient developed ocular motility impairment. Ophthalmology consultation considered that extraocular muscle involvement was likely attributable to ICI-associated inflammatory myopathy. However, orbital MRI and muscle biopsy were not performed. Therefore, this manifestation was described as ocular motility impairment rather than confirmed ocular myositis. Testing for anti-AChR, anti-MuSK and anti-LRP4 antibodies, as well as mediastinal MRI, was not completed; therefore, concomitant neuromuscular junction involvement could not be completely excluded.

Taken together, the temporal association with QL1706 administration, progressive muscle weakness, dyspnoea, marked CK elevation, pulmonary function findings suggestive of severe restrictive ventilatory dysfunction, and electrophysiological evidence of myopathy supported the clinical diagnosis of QL1706-associated severe immune-related inflammatory myopathy with suspected respiratory muscle involvement.

**Figure 1:**
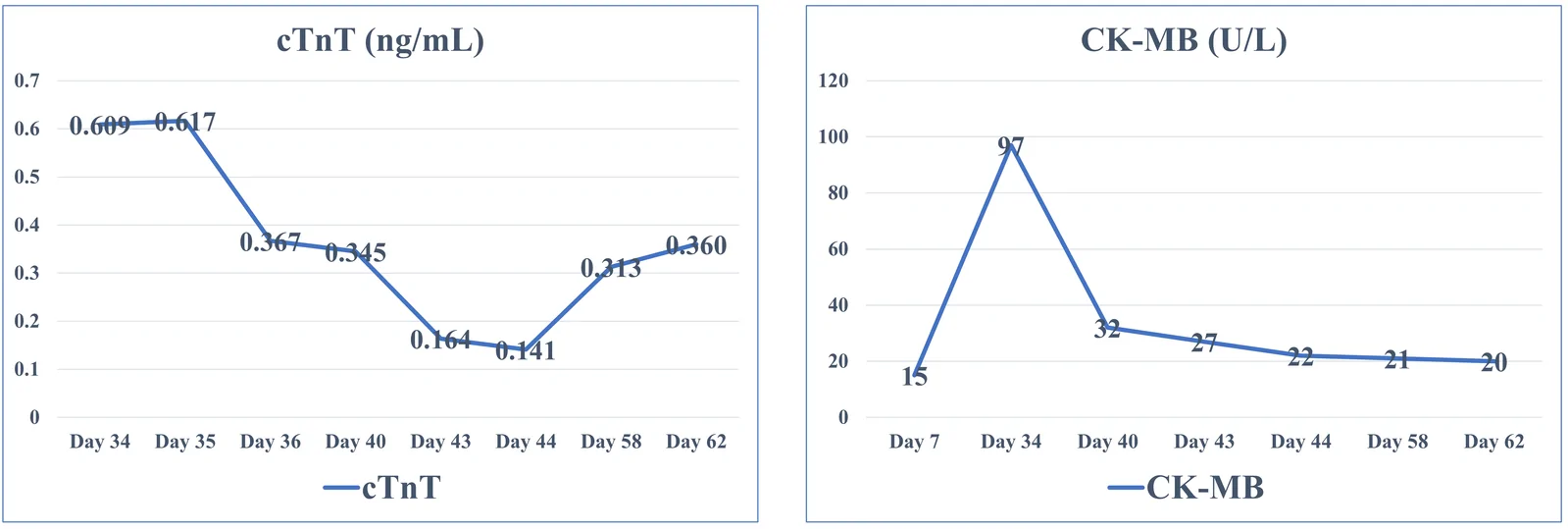
Temporal changes in cardiac-associated biomarkers after QL1706 administration. The first QL1706 administration was defined as Day 0. Serial changes in cardiac troponin T (cTnT) and creatine kinase-MB isoenzyme (CK-MB) are shown during the clinical course. The institutional reference range was < 0.014 ng/mL for cTnT and 0–24 U/L for CK-MB.

**Figure 2:**
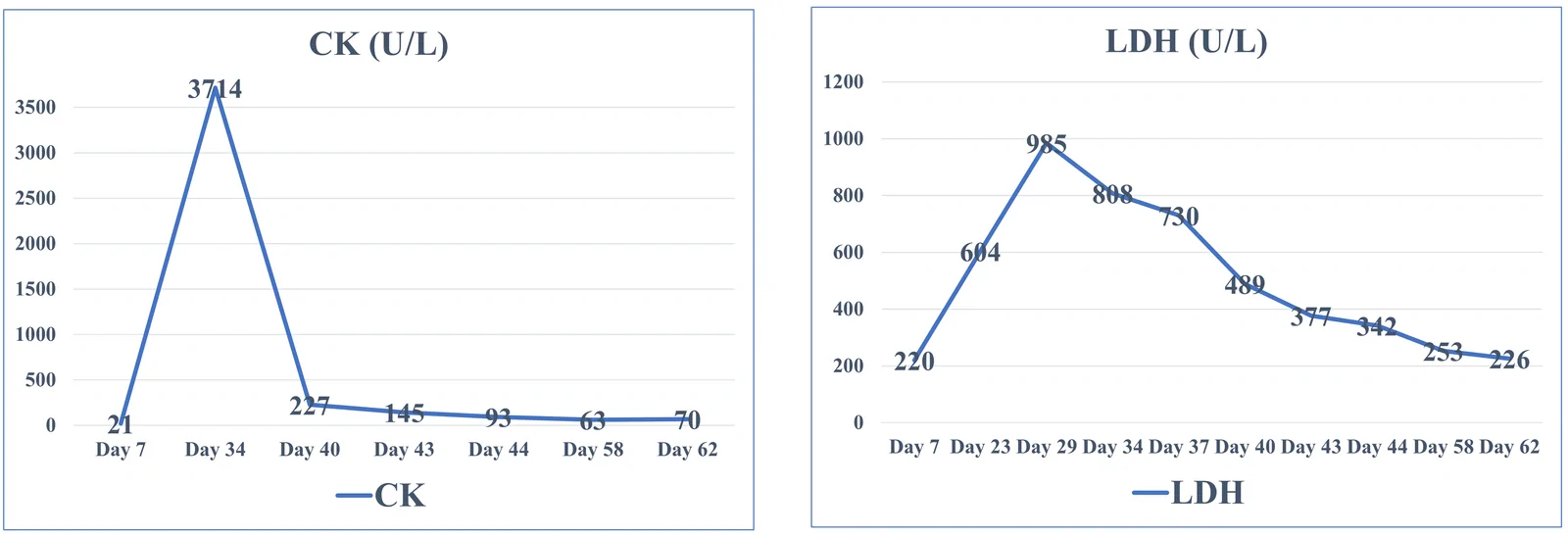
Temporal changes in muscle enzyme and lactate dehydrogenase after QL1706 administration. The first QL1706 administration was defined as Day 0. Serial changes in creatine kinase (CK) and lactate dehydrogenase (LDH) are shown during the clinical course. The institutional reference range was < 164 U/L for CK in females and 120–250 U/L for LDH.

**Figure 3:**
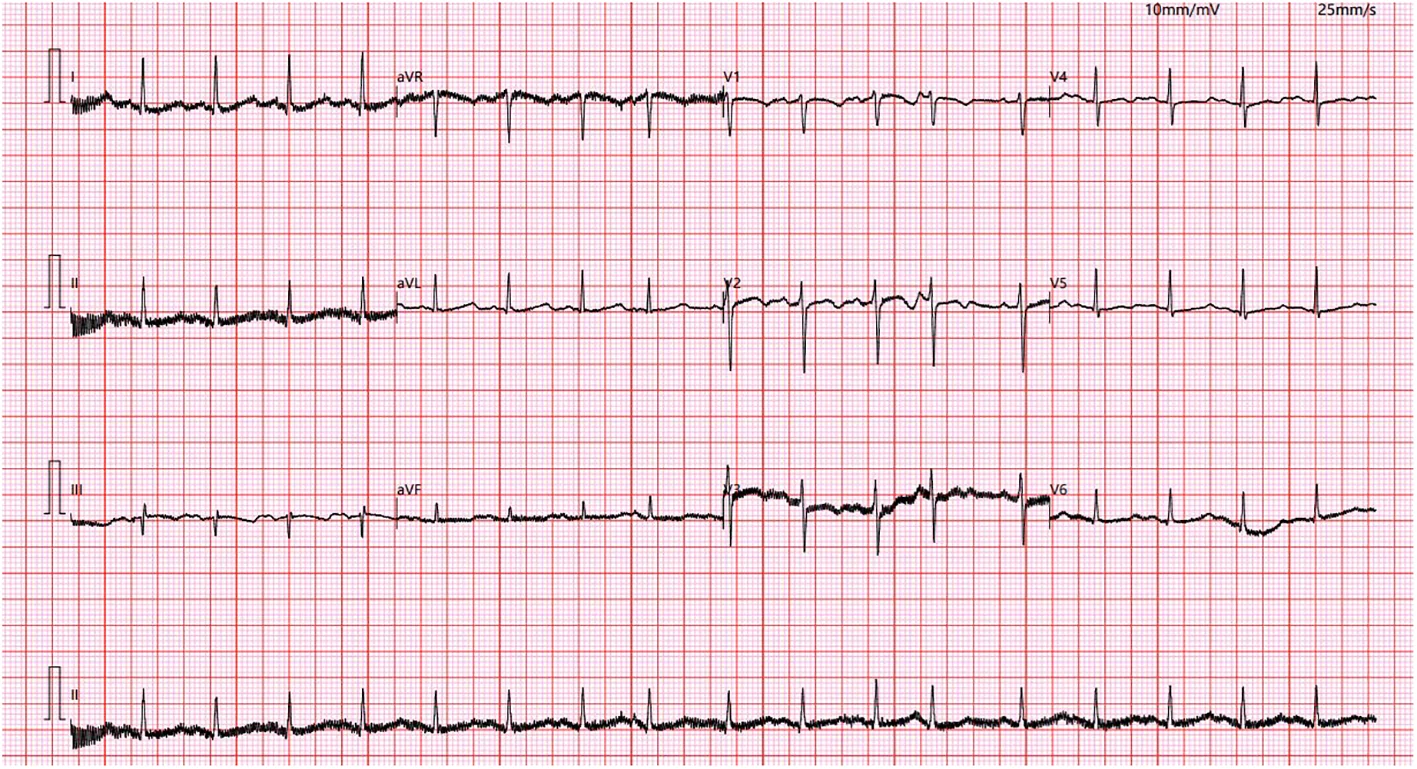
12-lead electrocardiogram obtained during the initial symptomatic phase. Diagnostic findings: (1) Sinus tachycardia; (2) Premature atrial contractions; (3) T-wave abnormalities; (4) Prolonged QT interval.

**Figures 1** and **2** show the serial changes in cTnT, CK-MB, CK and LDH during the treatment course. LDH peaked earlier than CK and declined relatively slowly, which may partly reflect its longer serum half-life. **Figure 3** shows the 12-lead ECG obtained at disease onset.

### Management and outcome

Management was guided by multidisciplinary consensus. Intravenous methylprednisolone was initiated promptly after immune-related inflammatory myopathy was clinically suspected. Because of worsening orthopnoea and cardiopulmonary symptoms, the patient first received methylprednisolone 500 mg/day as pulse therapy for 3 days, followed by gradual tapering after initial improvement. After the subsequent development of ocular motility impairment, methylprednisolone was re-escalated to 500 mg/day as pulse therapy for another 3 days, combined with intravenous immunoglobulin (IVIG; total dose, 2 g/kg over 5 days) as adjunctive immunomodulatory therapy. Tacrolimus, a calcineurin inhibitor, was also introduced as a steroid-sparing immunosuppressive agent, with a target trough concentration of 5–10 ng/mL. Supportive measures included high-flow nasal oxygen, individualised fluid-balance monitoring to avoid volume overload while maintaining adequate perfusion, and antimicrobial prophylaxis.

The patient responded favourably to this intensive immunosuppressive regimen. A gradual corticosteroid taper was then initiated, with transition from intravenous methylprednisolone to oral dexamethasone. Her cardiac and respiratory symptoms improved substantially, and follow-up electrocardiography returned to normal. Mild residual proximal lower-limb weakness persisted. The patient was discharged after 35 days on low-dose dexamethasone and tacrolimus, with close monitoring for recurrence of immune-related adverse events.

## Discussion

This case highlights severe immune-related inflammatory myopathy occurring shortly after QL1706-based treatment, with suspected respiratory and cardiac involvement and ocular motility impairment. QL1706 is a dual PD-1/CTLA-4 blockade product designed to enhance antitumour immune activation while modulating systemic toxicity (4). However, dual checkpoint inhibition remains biologically capable of inducing severe irAEs. Previous reports of combined PD-1 and CTLA-4 blockade, particularly nivolumab plus ipilimumab, have described severe myositis, myocarditis and myasthenia gravis overlap syndromes, supporting the need for vigilance when neuromuscular symptoms occur after dual-checkpoint therapy (8, 9).

The diagnosis in the present case was most consistent with QL1706-associated immune-related inflammatory myopathy. This was supported by the close temporal association with QL1706 administration, progressive weakness and myalgia, marked CK elevation, electrophysiological evidence of myopathy and improvement after immunosuppressive treatment. The term “polymyositis” was avoided because ICI-associated myositis has clinical features distinct from idiopathic inflammatory myopathies and may overlap with myocarditis or myasthenia gravis (8, 10). Together with previous QL1706-related reports, the present case suggests that severe neuromuscular toxicity may represent an important component of the immune-related toxicity spectrum of this PD-1/CTLA-4 bifunctional antibody (6, 7).

Cardiac involvement in this patient should be interpreted cautiously. Chest tightness, dyspnoea, ECG abnormalities and elevated CK-MB/cTnT raised clinical suspicion of cardiac involvement. However, cTnI was not measured and cardiac MRI was not performed. Although echocardiography showed preserved left ventricular systolic function without significant regional wall-motion abnormalities, these findings were insufficient to exclude myocarditis (11). In addition, the CK-MB/CK ratio on Day 34 was 2.61%, which may support a substantial skeletal muscle contribution to CK-MB elevation in the setting of severe inflammatory myopathy. cTnT elevation may also be confounded by skeletal muscle injury in active inflammatory myopathy, whereas cTnI is generally considered more specific for myocardial injury in this context (12). Therefore, the cardiac findings were described as clinically suspected cardiac involvement rather than confirmed immune-related myocarditis.

The ocular, respiratory and bulbar manifestations also required cautious interpretation. Ocular involvement is a recognised but frequently misdiagnosed manifestation of ICI-associated myositis, and orbital imaging may help identify suspected extraocular muscle involvement (13). In the present case, the patient developed ocular motility impairment, which was considered by the ophthalmology team to be possibly related to extraocular muscle involvement. However, orbital MRI and muscle biopsy were not performed; therefore, extraocular myositis could not be definitively diagnosed. Pulmonary function testing showed marked reductions in vital capacity, forced vital capacity, and maximal voluntary ventilation, with a preserved FEV1/FVC ratio, and was interpreted as demonstrating a severe restrictive ventilatory defect. Liver MRI also incidentally revealed abnormal signal within the thoracic musculature. Together with progressive overall weakness and dyspnoea, these findings supported suspected respiratory muscle involvement. However, diffusing capacity could not be assessed because of limited patient cooperation, and respiratory muscle strength and diaphragmatic function were not directly evaluated; therefore, respiratory muscle involvement could not be definitively confirmed. The presence of dysphagia and dysarthria supported clinical bulbar weakness, although its underlying mechanism—whether related to inflammatory myopathy or concomitant neuromuscular junction involvement remained uncertain (14).

ICI-associated myositis, myocarditis, and myasthenia gravis may occur as a myositis–myocarditis–myasthenia gravis overlap syndrome, which is associated with substantial mortality (8, 9, 15). In this case, repetitive nerve stimulation showed no significant abnormality; however, anti-AChR, anti-MuSK, and anti-LRP4 antibodies were not tested, and single-fibre electromyography was not performed. Therefore, concomitant neuromuscular junction involvement could not be completely excluded. Nevertheless, because neither myocarditis nor myasthenia gravis was definitively established, the criteria for a confirmed MMM overlap syndrome could not be established.

Treatment was guided by multidisciplinary consensus. The overall strategy of ICI interruption, high-dose intravenous corticosteroids, IVIG, escalation of immunosuppression, and supportive respiratory care was broadly consistent with ASCO and ESMO recommendations for severe neuromuscular irAEs (16, 17). The patient’s clinical improvement supported an immune-mediated mechanism, although causality cannot be established from a single case. Any future ICI rechallenge should be considered on a case-by-case basis following multidisciplinary risk–benefit assessment; this case does not provide sufficient evidence to support a specific rechallenge strategy.

This report has several limitations. cTnI testing, cardiac MRI, orbital MRI, dedicated skeletal muscle MRI, muscle or myocardial biopsy, myasthenia gravis-related antibody testing, and single-fibre electromyography were not performed. Respiratory muscle strength and diaphragmatic function were also not directly assessed. Accordingly, myocarditis, extraocular myositis, respiratory muscle involvement, and concomitant neuromuscular junction involvement could not be definitively confirmed, and a confirmed MMM overlap syndrome could not be established.

## Conclusion

This case suggests that severe immune-related inflammatory myopathy can occur within the first month after QL1706 treatment and may present with respiratory dysfunction, bulbar weakness, and ocular motility impairment, together with clinically suspected cardiac involvement.

## Data Availability

The original contributions presented in the study are included in the article. Further inquiries can be directed to the corresponding author.
